# Nectar-Inhabiting Bacteria Affect Olfactory Responses of an Insect Parasitoid by Altering Nectar Odors

**DOI:** 10.1007/s00248-022-02078-6

**Published:** 2022-08-01

**Authors:** Antonino Cusumano, Patrizia Bella, Ezio Peri, Michael Rostás, Salvatore Guarino, Bart Lievens, Stefano Colazza

**Affiliations:** 1 Department of Agricultural, Food and Forest Sciences, University of Palermo Viale delle Scienze, Building 5, 90128 Palermo, Italy; 2 Interuniversity Center for Studies on Bioinspired Agro-Environmental Technology (BATCenter), University of Napoli Federico II, 80055 Portici, Italy; 3 Agricultural Entomology, Department of Crop Sciences, University of Göttingen, Grisebachstr. 6, 37077 Göttingen, Germany; 4 Institute of Biosciences and Bioresources (IBBR), National Research Council of Italy (CNR), Corso Calatafimi 414, 90129 Palermo, Italy; 5 Laboratory for Process Microbial Ecology and Bioinspirational Management (PME&BIM), Department of Microbial and Molecular Systems, Willem De Croylaan 46, Leuven, KU 3001 Belgium; 6 Leuven Plant Institute (LPI), Leuven, KU 3001 Belgium

**Keywords:** Nectar-associated microbes, Parasitoid foraging behavior, Conservation biological control, *Fagopyrum esculentum*, *Trissolcus basalis*

## Abstract

Floral nectar is ubiquitously colonized by a variety of microorganisms among which yeasts and bacteria are the most common. Microorganisms inhabiting floral nectar can alter several nectar traits, including nectar odor by producing microbial volatile organic compounds (mVOCs). Evidence showing that mVOCs can affect the foraging behavior of insect pollinators is increasing in the literature, whereas the role of mVOCs in altering the foraging behavior of third-trophic level organisms such as insect parasitoids is largely overlooked. Parasitoids are frequent visitors of flowers and are well known to feed on nectar. In this study, we isolated bacteria inhabiting floral nectar of buckwheat, *Fagopyrum esculentum* (Polygonales: Polygonaceae), to test the hypothesis that nectar bacteria affect the foraging behavior of the egg parasitoid *Trissolcus basalis* (Hymenoptera: Scelionidae) via changes in odors of nectar. In behavioral assays, we found that *T*. *basalis* wasps are attracted toward nectar fermented by 4 out of the 14 bacterial strains isolated, which belong to *Staphylococcus epidermidis*, *Terrabacillus saccharophilus* (both Firmicutes), *Pantoea* sp. (Proteobacteria), and *Curtobacterium* sp. (Actinobacteria). Results of chemical investigations revealed significant differences in the volatile blend composition of nectars fermented by the bacterial isolates. Our results indicate that nectar-inhabiting bacteria play an important role in the interactions between flowering plants and foraging parasitoids. These results are also relevant from an applied perspective as flowering resources, such as buckwheat, are largely used in agriculture to promote conservation biological control of insect pests.

**Supplementary Information:**

The online version contains supplementary material available at 10.1007/s00248-022-02078-6.

## Introduction

Floral nectar is a sugar-rich resource that mainly consists of mono- and disaccharides, whereas amino acids, lipids, and vitamins are present in lower amounts [7, 45, 46]. Although the role of nectar has been intensively studied in interactions between flowering plants and pollinators, it is well known that other animals, such as hymenopteran parasitoids, also commonly visit flowers and feed on nectar [31]. Parasitoids are important components of terrestrial foodwebs and play a crucial role in biological control programs by regulating insect pest populations [29]. Similarly to pollinating insects, adult parasitoids need sugar resources in order to satisfy their energetic requirements, particularly when engaging in demanding behavioral activities such as searching for hosts [6, 58]. Thus, besides pollinators, insect parasitoids represent ideal candidates for studying how floral nectar affects the foraging behavior of flower-visiting insects.

Floral nectar is ubiquitously colonized by a variety of microorganisms among which yeasts and bacteria are the most frequently reported [50, 62]. In recent years, it has been shown that flower-associated microbes can modify the physical and chemical properties of nectar and other floral structures, and hence they should be considered important “third players” in the interactions between plants and flower-visiting insects [34, 40, 49, 65]. For example, it has been shown that nectar-inhabiting microbes can cause a shift in the sugar profile of floral nectar under field conditions where the sucrose-dominated nectar originally produced by the plant was modified toward a fructose-rich nectar [11, 30]. As a result of the metabolic activity of microorganisms in nectar, the quality of this sugar-rich resource may drastically change, not only in terms of sugar profile and concentration, but also in terms of amino acid composition and concentration, and pH [42, 50]. Furthermore, nectar-inhabiting microorganisms have been found to produce volatile organic compounds (mVOCs, microbial volatile organic compounds) which affect the scent of floral nectar [26, 51, 56] and, consequently, insect attraction to flowering plants [14]. In fact, there is plenty of evidence showing that mVOCs from nectar microbes can affect the foraging behavior of pollinators such as honeybees, bumblebees, and hummingbirds [27, 51–54, 65]. Nevertheless, the role played by mVOCs in parasitoid’s olfactory responses to floral nectar has often been overlooked. In fact, the only case study reported so far refers to the aphid parasitoid *Aphidius ervi* (Hymenoptera: Braconidae) which has been shown to be attracted by mVOCs emitted from nectar fermented by nectar specialist yeasts in the phylum Ascomycota such as *Metschnikowia reukaufii* and *M*. *gruessii* [56]. However, how parasitoids respond to nectar scent when fermented by bacteria is largely unknown.

In this study, we aim to fill this gap by investigating how bacteria associated with floral nectar of buckwheat (*Fagopyrum esculentum*) (Polygonales: Polygonaceae) affect olfactory responses of the egg parasitoid *Trissolcus basalis* (Hymenoptera: Scelionidae) via changes in floral nectar odors. *Trissolcus basalis* is the main biological control agent of the cosmopolitan and polyphagous stink bug pest *Nezara viridula* [15, 32]. In olfactometer experiments where *T*. *basalis* was allowed to choose between flowering and non-flowering plants, it was found that female wasps are strongly attracted to buckwheat flowers [22]. Positive olfactory responses of insect parasitoids to buckwheat are particularly interesting as this flowering plant species is widely used in conservation biological control programs [28, 36]. In fact, buckwheat flowers offer high quality and easily accessible nectar, which enhances the performance of several parasitoid species [23, 38, 39, 44]. Access to buckwheat flowering plants increases *T. basalis* fecundity in the laboratory [22] and parasitism rates of *N*. *viridula* eggs in the field [21]. Yet, despite the importance of buckwheat for biological pest control, no studies so far have characterized the microbial composition of buckwheat floral nectar. Consequently, the role of nectar-inhabiting microbes in mediating parasitoid attraction to buckwheat flowering plants also remains to be investigated. Nonetheless, considering that mVOCs emitted by pure cultures of bacteria associated with parasitoid habitats affect parasitoid foraging behavior [24], it is reasonable to assume that parasitoids should be able to perceive and respond to odors of bacteria-fermented nectar.

In this work, we first isolated and characterized the culturable bacteria associated with buckwheat floral nectar. Next, we investigated the bacteria-mediated effects in terms of parasitoid attraction to fermented nectar. Finally, we analyzed the chemical nature of mVOCs to explain which compounds could mediate parasitoid olfactory responses.

## Material and Methods

### Plant Rearing and Nectar Collection

Seeds of buckwheat (*F*. *esculentum* cv kaitowase) were sown in 10-cell plug trays using commercial potting mix “Supernutrient Vegetable Soil” (Vigorplant, Piacenza, Italy). Trays were put in a climate-controlled chamber (24 ± 2 °C, 45 ± 10% RH, 12 h:12 h L:D). After germination, 1-week-old seedlings were transplanted into 1 L plastic pots with the same type of potting mix. After one additional week, plants were placed at the beginning of each month during May–October 2020 in the experimental fields of the University of Palermo and exposed to the natural community of insects and microbes present in the area to maximize the diversity of microbes that can be sampled on floral nectar, and which may change over time. The first week of each month, floral nectar was sampled from plants that were approximately 4–5 weeks old. On the day of nectar collections, plants were transported to the laboratory early in the morning where nectar was collected from fully opened flowers. Nectar was sampled under sterile conditions in a laminar flow cabinet with the aid of 0.5 µL glass capillary tubes (see Cawoy et al. [12] for a detailed description of the sampling procedure) and transferre to 1.5 mL Eppendorf tubes previously filled with 100 µL of sterile demineralized water. In each tube, nectar from 50 flowers (10 flowers/plant) was collected (~ 0.05 µL of nectar/flower), and a total of 20 samples were prepared for isolation of nectar bacteria.

### Bacterial Isolation from Buckwheat Floral Nectar

Isolation of bacteria was carried out immediately after nectar collection by plating 100 µL of the sample on trypticase soy agar (TSA) (Oxoid, Basingstoke, UK). Plates were incubated at 27 °C for 5–7 days. For each plate, distinct morphotypes were sub-cultivated on TSA to obtain pure cultures. For long term storage, stock cultures of bacteria were preserved in trypticase soy broth (TSB) plus 40% glycerol at – 80 °C.

For taxonomic identification, the 16S ribosomal RNA (rRNA) gene region was amplified using the universal primer pair 27F and 1492R [18, 37]. Bacterial DNA was prepared by thermal lysis of cell suspensions in 200 μL of sterile distilled water at 100 °C for 10 min. All amplifications were performed in a final volume of 25 μL containing 1 × Taq Go® G2 Hot Start Colorless PCR Master Mix (Promega), 1 μM of each primer, and 1 µL of DNA template. Reactions were performed in a MultiGene Optimax Gradient thermal cycler (Labnet International snc) by using the PCR cycling conditions previously reported [5]. The PCR products were subjected to electrophoresis on 1.0% agarose gel, quantified and sent to BMR Genomics (Padova, Italy) for sequencing using the reverse primer used for PCR amplification. The sequences were then compared with reference type materials from GenBank using Basic Local Alignment Search Tool BLASTN (http://www.ncbi.nlm.nih.gov) and assigned to the lowest reliable taxonomic rank possible based on highest sequence homology with GenBank entries (species or genus) (see Online Resource 1 of the Electronic Supplementary Material, ESM). Furthermore, a phylogenetic analysis was performed to visualize relationships between our isolates and the most related type strains found in GenBank. Therefore, our sequences and a number of reference sequences downloaded from GenBank were aligned (~ 800 bp) with Clustal W implemented in MEGA-X [35]. Neighbor-joining (NJ) trees were generated with MEGA X using the Jukes–Cantor distances method and 1000 bootstrap replicates (Online Resource 2, ESM). Sequences obtained in this study have been deposited in GenBank under the accession numbers from ON166769 to ON166782.

For bacterial isolates never found in floral nectar in previous studies, phenotypic characterization of bacterial isolates was carried out by assessing their catalase activity, sucrose tolerance, and the ability to grow at low oxygen levels (microaerobiosis), according to Alvarez-Perez et al. [4].

### Parasitoid Rearing

A colony of *T*. *basalis* was established from wasps emerging from sentinel *N*. *viridula* egg masses placed in tomato fields infested by *N*. *viridula* in Palermo, Italy. Parasitoids were reared in 16-mL glass tubes (density = 70–100 wasps/tube), fed with a 50/50% honey–water solution, and kept in a climate chamber (24 ± 2 °C, 80 ± 5% RH, 16 h:8 h L:D). To maintain the insects, freshly collected egg masses from a laboratory culture of *N*. *viridula* were bi-weekly exposed to 2–3 parasitoid females for 24 h, then the eggs were removed and stored for incubation. After emergence, male and female parasitoids were kept together to allow for mating. In all bioassays, *T*. *basalis* females were used when they were 4–5 day old. About 24 h before the experiments, wasp females were individually put in small vials (1.5 × 5 cm) without food to induce starvation.

The laboratory culture of *N*. *viridula* was originally established from field collected bugs in cultivated and noncultivated fields located around Palermo. Insects were held in wooden cages (50 × 30 × 35 cm) provided with mesh-covered holes for ventilation (5 cm in diameter), in a climate chamber (24 ± 1 °C, 70 ± 5% RH, 16 h:8 h L:D). Bugs were fed with a diet of organic seasonal vegetables and raw sunflower seeds. Separate cages were used for nymphs and adults. Daily collected egg masses were used to maintain the laboratory colony which was also regularly often supplemented with new field collected bugs.

### Synthetic Nectar Solutions

Synthetic nectar was prepared by filter-sterilizing 50% w/v sucrose solution supplemented with 3.16 mM amino acids from digested casein [63, 64]. Synthetic nectar was then fermented with individual bacterial isolates as described by Lenaerts et al. [40] in order to prepare test solutions for olfactometer investigations and GC–MS analyses. Briefly, single colonies from 48-h bacterial cultures in TSA were inoculated into 10 mL of TSB and incubated overnight at 25 °C on a rotary shaker at 150 rpm. After that, cells were washed two times and suspended in sterile physiological water (0.9% NaCl) and concentration-adjusted to an optical density of 1 (OD600) (about 10^8^ cfu/mL). Next, 10 mL of synthetic nectar was inoculated with 100 µL bacterial suspension (representing a concentration of 10^3^ cfu/µL, which is in line with field observations [3, 60, 65] and incubated at 25 °C for 5 days to prepare test solutions whereas non-inoculated synthetic nectar was used as negative control. The sterility of the negative control was checked by plating on TSA. To obtain cell-free cultures, inoculated and non-inoculated synthetic nectar solutions were filtered (pore size 0.2 μm,Exacta + Optech Labcenter SpA, Italy) and stored in glass amber vials in aliquots of 1 mL at – 80 °C. All fermentations were performed in five biological replicates which were carried out across different days using a randomized experimental design.

### Bacterial Effects on Parasitoid Olfactory Responses Toward Nectar Odors

The olfactory response of *T*. *basalis* to the synthetic nectar fermented by the bacterial isolates was tested in a four-chamber static olfactometer device [22, 57]. Briefly, the olfactometer consisted of an acrylic glass body cylinder (4.5 cm high and 20 cm diameter) divided equally into four chambers which were closed on top by a removable, gauze-covered (mesh: 0.5) walking arena (1.5 cm high and 20 cm diameter). The following bioassays were performed: (A) diluted buckwheat raw nectar (2.5% v/v) versus non-fermented synthetic nectar; (B) distilled water versus non-fermented synthetic nectar; and (C) synthetic nectar fermented by the individual bacterial isolates versus non-fermented synthetic nectar. In the experiments, a standard volume of 100 µL of diluted raw nectar (bioassay A), or distilled water (bioassay B), or cell-free fermented synthetic nectar (bioassay C), and non-fermented synthetic nectar (bioassay A, B, and C) was pipetted on a filter paper disk (Whatman No. 1) fitted inside a Petri dish (5 cm diameter). An odor sample of the same type (raw nectar, distilled water, or synthetic nectar fermented by the bacterial isolates) was placed in two opposite chambers, whereas non-fermented synthetic nectar (control odor) was placed in the other two chambers. A single female parasitoid, acclimatized for at least 1 h in the bioassay room before the experiment, was released in the center of the walking arena and allowed to explore the arena for 1 min. Subsequently, the time the wasp spent above each odor chamber (residence time) was recorded for 5 min with the aid of JWatcher V 0.9 software [9], https://www.jwatcher.ucla.edu/. Preliminary observations without odor sources indicated that there were no positional biases in the set-up. During the experiments, the positions of the test and control samples were switched after each observation to correct for any unforeseen positional bias in the set-up. At the end of the day, the device was cleaned using 70% ethanol, rinsed with distilled water, and left to dry at room temperature. The experimental set-up was surrounded with black curtains to avoid visual cues, and the olfactometer was illuminated from above by two cool white fluorescent tubes (Philips, TLD 58 W/640). Experiments were conducted from 8:30 to 14:00 h and the temperature in the bioassay room was 24 ± 1 °C. Twenty wasp females were tested for each pairwise comparison using a full randomized experimental design. As the VOC composition of all five biological replicates of nectar was highly similar (see results), olfactory response was determined for one of the five biological replicates.

### Bacterial Effects on the Chemistry of Nectar Odors

For each biological replicate (five per treatment), VOCs were collected with head space-solid phase micro extraction (HS-SPME) technique, using Carbowax–divinylbenzene (CW-DVB, 65 μm) fiber (Supelco, Bellefonte, PA, USA) as stationary phase, and a manual SPME holder from the same manufacturer for injections. Fibers were conditioned in a gas chromatograph injector port at 220 °C for 30 min as recommended by the manufacturer. In all analyses,100 µL of cell-free fermented synthetic nectar (or non-fermented synthetic nectar as control) were pipetted on a filter paper disk (5 cm diameter), which was then placed into a 40-mL vial sealed with a poly(tetrafluoroethylene) silicon septum-lined cap (Supelco, Bellefonte, PA, USA). Five minutes later, the SPME needle was inserted through the septum and headspace volatiles were absorbed on the exposed fiber for 1 h. The loaded fiber was then desorbed in the gas chromatograph-mass spectrometer (GC–MS) inlet port for 1 min. Chemical analyses were performed using an Agilent 6890 GC system equipped with a DB5-MS column and interfaced with an MS5973 quadruple mass spectrometer. The GC–MS was set in spitless mode with helium used as carrier gas. Injector and detector temperatures were 260 °C and 280 °C, respectively. The GC oven temperature was set at 40 °C and then increased by 10 °C/min to 250 °C, with initial and final hold times of 5 and 30 min, respectively. Electron impact ionization spectra were obtained at 70 eV, recording mass spectra from 40 to 550 amu. For quantification purposes, peak area of each detected compound was calculated using ChemStation software. Compounds were tentatively identified based on comparison of retention index and mass spectra with those reported in the literature [1], www.pherobase.com and the NIST 2011 and Wiley 17 libraries, and by injection of authentic standards when available (Sigma-Aldrich, Milan, Italy). Identification was assumed when a good match of mass spectrum and RI was achieved. To exclude from the analysis the presence of possible contaminants, blank headspace collections were carried out periodically.

### Statistical Analyses

Residence time data were not significantly different from a normal distribution (Shapiro–Wilk test) and thus analyzed with parametric tests. A paired *t* test for dependent samples was used to process the total residence time spent by the wasps in the four chamber olfactometer comparing the time spent by the wasp on the two test chambers (containing the same odor type) versus the time spent in the control chambers (always containing non-fermented synthetic nectar). Multivariate data analysis (projection to latent structures discriminant analysis—PSL-DA) was used to analyze peak areas of chemical compounds. The measured peak areas were first log-transformed, mean-centered and subsequently scaled to unit variance before they were subjected to the analysis using the software MetaboAnalyst [69]. The results of the analysis were visualized in score plots, which reveal the sample structure according to model components, and loading plots, which display the contribution of the variables to these components. The ranking of the compounds that contribute the most in explaining statistical differences were identified based on the variable importance in the projection (VIP values) [68].

## Results

### Bacterial Isolation from Buckwheat Floral Nectar

Bacterial populations recovered from buckwheat floral nectar ranged from 2.1 to 3.5 Log10 colony-forming units (cfu)/mL. In total, 14 different morphotypes were found and identified by partially sequencing the 16S rRNA gene and subsequent phylogenetic analysis. Isolated strains belonged to three phyla, including Firmicutes (8 isolates), Proteobacteria (4 isolates), and Actinobacteria (2 isolates) (Online Resource 1, ESM). Within the Firmicutes, four isolates belonged to the family Bacillaceae (*Bacillus* sp. SAAF 22.2.6, *Bacillus* sp. SAAF 22.2.27, and *Brevibacterium frigoritolerans* SAAF 22.2.4 and *Terribacillus saccharophilus* SAAF 22.2.3), whereas the other four isolates were identified in the family Paenibacillaceae (*Brevibacillus* sp. SAAF 22.4.13 and *Saccharobacillus* sp. SAAF 22.4.25) or in the family Staphylococcaceae (*Staphylococcus epidermidis* SAAF 22.3.11 and *Staphylococcus hominis* SAAF 22.3.10). All four isolates within Proteobacteria belonged to the family Erwiniaceae (*Pantoea agglomerans* SAAF 22.4.2, *Pantoea dispersa* SAAF 22.3.3, and *Pantoea* sp. SAAF 22.4.17). For Actinobacteria, we isolated, in the family Microbacteriaceae, *Curtobacterium* sp. SAAF 22.3.18 and, in the family Promicromonosporaceae, *Cellulosimicrobium* sp. SAAF 22.3.25. The phylogenetic relationships between the isolated bacteria inhabiting buckwheat floral nectar and a number of closely related reference strains are visualized in the phylogenetic tree displayed in the Online Resource 2, reinforcing the taxonomic classification of our isolates. The three bacterial isolates (*Cellulosimicrobium* sp. SAAF 22.3.25, *Brevibacillus* sp. SAAF 22.4.13 and *Saccharibacillus* sp. SAAF 22.4.25) that, to our knowledge, were recovered for the first time in floral nectar were able to grow under microaerobiosis, showed a positive reaction in the catalase test and tolerated 10–30% (w/v) sucrose (data not shown).

### Bacterial Effects on Parasitoid Olfactory Responses Toward Nectar Odors

**Fig. 1:**
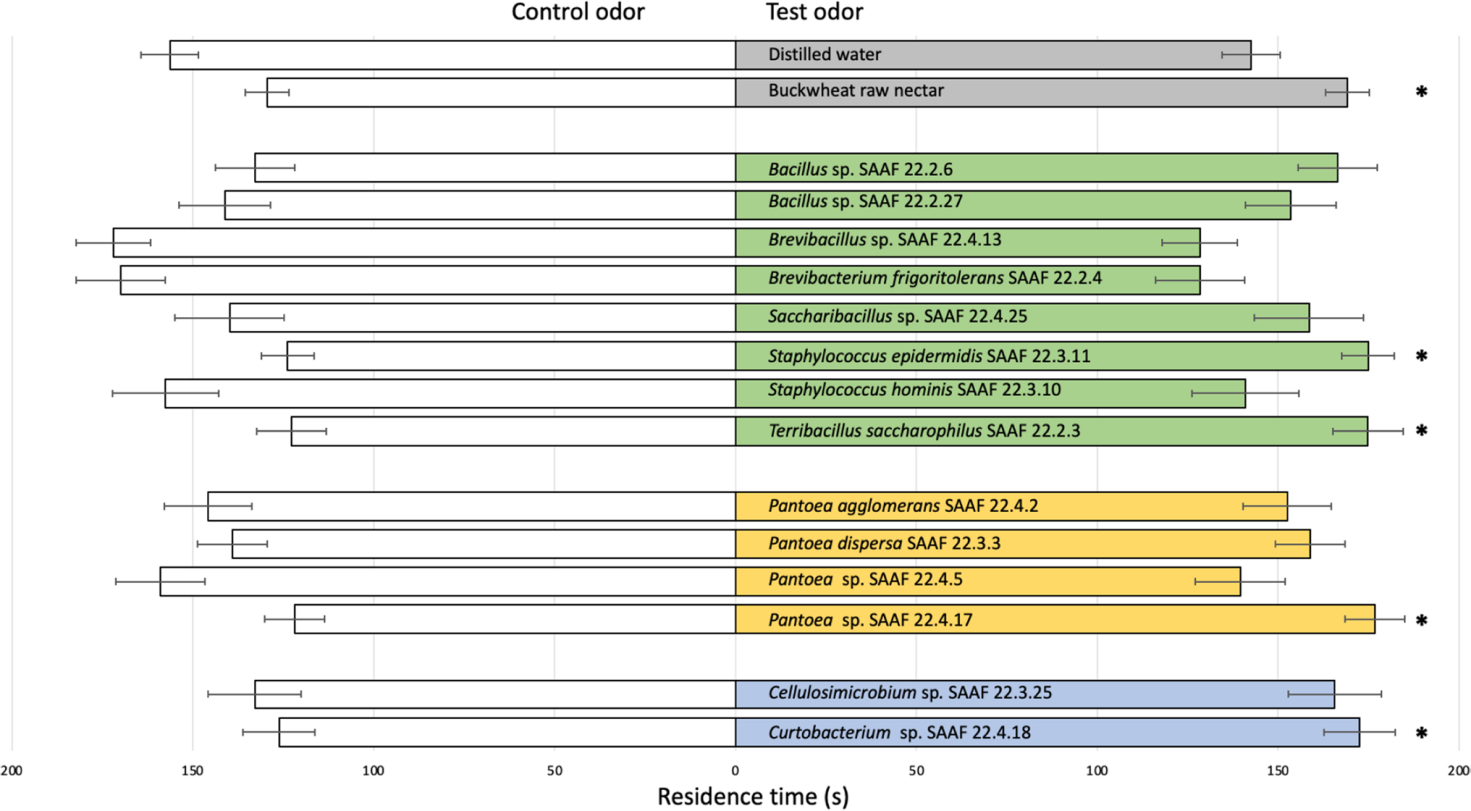
Olfactory response of adult *Trissolcus basalis* females when given the choice between test and control odors. Test odors included buckwheat raw nectar or synthetic nectar fermented by: for **Firmicutes—***Bacillus* sp. SAAF 22.2.6, *Bacillus* sp. SAAF 22.2.27, *Brevibacillus frigoritolerans* SAAF 22.2.4, *Brevibacillus* sp. SAAF 22.4.13, *Saccharobacillus* sp. SAAF 22.4.25, *Staphylococcus epidermidis* SAAF 2.3.11, *Staphylococcus hominis* SAAF 22.3.10, and *Terribacillus saccharophilus* SAAF 22.2.3; for **Proteobacteria**—*Pantoea agglomerans* SAAF 22.4.2, *Pantoea dispersa* SAAF 22.3.3, *Pantoea* sp. SAAF 22.4.17, and *Pantoea* sp SAAF 22.4.5; for **Actinobacteria**—*Curtobacterium* sp. SAAF 22.3.18 and *Cellulosimicrobium* sp. SAAF 22.3.25. The control in all pairwise comparisons was non-fermented synthetic nectar (white bars). All experiments were performed with cell-free nectars. Bars indicate mean (± SE) of the time spent by wasp females in test or control chambers over an observation period of 300s. Grey bars = distilled water and Buckwheat raw nectar, Green bars = Firmicutes, Yellow bars = Proteobacteria, Blue bars = Actinobacteria. Each experiment was replicated 20 times (paired *t* tests, *P ≤ 0.05; *NS* non-significant)

*Trissolcus basalis* females significantly preferred the odors emitted by buckwheat raw nectar over the odors associated with non-fermented synthetic nectar (*t* = 3.284, df = 19, *P* = 0.004), whereas no significant differences were found between odors of non-fermented synthetic nectar and distilled water (*t* =  − 0.544, df = 19, *P* = 0.592). When nectar fermented by Firmicutes bacteria was tested against non-fermented synthetic nectar, a significant attraction was found for *T*. *saccharophilus* SAAF 22.2.3 (*t* = 2.691, df = 19, *P* = 0.014) and *S*. *epidermidis* SAAF 2.3.11 (*t* = 3.536, df = 19, *P* = 0.002), whereas no differences were found for *Bacillus* sp. SAAF 22.2.6 (*t* = 1.532, df = 19, *P* = 0.142), *Bacillus* sp. SAAF 22.2.27 (*t* = 0.332, df = 19, *P* = 0.743), *B. frigoritolerans* SAAF 22.2.4 (*t* =  − 1.691, df = 19, *P* = 0.107), *Brevibacillus* sp. SAAF 22.4.13 (*t* =  − 2.018, df = 19, *P* = 0.057), *Saccharobacillus* sp. SAAF 22.4.25 (*t* = 0.624, df = 19 *P* = 0.539), and *S. hominis* SAAF 22.3.10 (*t* =  − 0.565, df = 19, *P* = 0.578). When nectar fermented by Proteobacteria was tested against non-fermented synthetic nectar, *T*. *basalis* females spent significantly more time on the olfactometer chambers containing nectar fermented by *Pantoea* sp. SAAF 22.4.17 (*t* = 3.301, df = 19, *P* = 0.003), whereas no response was elicited by *P*. *agglomerans* SAAF 22.4.2 (*t* = 0.278, df = 19, *P* = 0.783), *P*. *dispersa* SAAF 22.3.3 (*t* = 1.037, df = 19 *P* = 0.313), and *Pantoea* sp. SAAF 22.4.5 (*t* =  − 0.778, df = 19, *P* = 0.445). When nectar fermented by Actinobacteria was tested against non-fermented synthetic nectar, parasitoids preferred odors associated with *Curtobacterium* sp. SAAF 22.4.18 (*t* = 2.332, df = 19, *P* = 0.031), but not odors emitted by *Cellulosimicrobium* sp. SAAF 22.3.25 (*t* = 1.271, df = 19 *P* = 0.219). Regardless of the bacteria species involved, parasitoids never preferred the non-fermented nectar over the fermented nectar (Fig. 1).

### Bacterial effects on the chemistry of nectar odors

A total of 27 different volatile organic compounds were detected in the headspace of nectar fermented by the different bacterial isolates, whereas a total of 24 compounds were found in non-fermented synthetic nectar. Overall, different isolates emitted the same compounds, but in different proportions with the exception of the compounds butanediol and 2,3-butanediol that were only detected in the headspace of *Bacillus* sp. SAAF 22.2.27, *P*. *agglomerans* SAAF 22.4.2, and *P*. *dispersa* SAAF 22.3.3. The compound 2,6-di-tert-butyl-*p*-benzoquinone was always detected in nectar samples fermented by bacterial isolates, but never in control samples (non-fermented synthetic nectar).

**Fig. 2:**
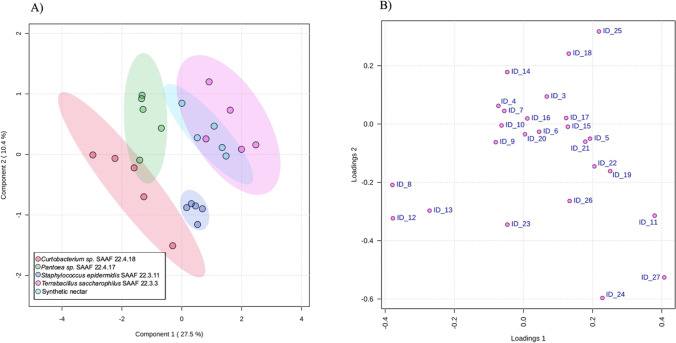
Projection to latent structures discriminant analysis (PLS-DA) of synthetic nectar fermented by the bacteria that elicited a significant olfactory attraction in the parasitoid *Trissolcus basalis*. Treatments included synthetic nectar fermented by *Curtobacterium* sp. SAAF 22.4.18, *Pantoea* sp. SAAF 22.4.17, *Staphylococcus epidermidis* SAAF 2.3.11, *Terribacillus saccharophilus* SAAF 22.2.3, and a negative control (non-fermented synthetic nectar). All biological replicates (*N* = 5) indicate cell-free nectars. **A** Score plot visualizing the grouping pattern of the samples according to the first two principal components (PCs) with the explained variance in parenthesis. Ellipses indicate 95% confidence interval. **B** Loading plot of the first two PCs showing the contribution of each compound to the two PLS-DA components. Variable important for the projection (VIP) with a value > 1.5 are ID 12 = 2-methoxy-*p*-cymene; ID 8 = dimethyl glutarate; ID 27 = dihydrojasmonate; ID 11 = 2,5-dymethylbenzaldehyde; ID13 = Unknown. See Table 1 for the full list of compound IDs

A comparison by PLS-DA among the four isolates of bacteria that elicited a significant attraction in the olfactometer (i.e., *Curtobacterium* sp. SAAF 22.4.18, *Pantoea* sp. SAAF 22.4.17, *S. epidermidis* SAAF 2.3.11, *T. saccharophilus* SAAF 22.2.3) and non-fermented synthetic nectar resulted in a significant model (permutation test, *P* < 0.001) (Fig. 2). The model largely separated volatiles emitted by the nectars fermented with the different bacteria in agreement with the behavioral observations. The model also shows a separation of control volatiles (emitted by non-fermented synthetic nectar) from those associated with fermentation by *S*. *epidermidis* SAAF 2.3.11, *Pantoea* sp. SAAF 22.4.17 and *Curtobacterium* sp. SAAF 22.4.18, while no separation was found between control volatiles and volatiles from nectar fermented with *T*. *saccharophilus* SAAF 22.2.3 (Fig. 2). In the PLS-DA model, five compounds had a VIP value > 1.5 indicating that these compounds strongly contributed to explaining the differences among treatments. These compounds were 2-methoxy-*p*-cymene, glutaric acid dimethyl ester, methyl dihydrojasmonate, 2,5-dimethylbenzaldehyde, and an unknown compound (Table 1).

**Table 1:** Volatile emissions^1^ of synthetic nectar fermented by bacteria isolated from buckwheat nectar

				**Phylum**													
				**Firmicutes**									**Proteobacteria**			**Actinobacteria**	
**RT (RI)**	**Chemicals**	**ID**^**b**^	**Synthetic nectar**	***Bacillus* 22.2.6**	***Bacillus* 22.2.27**	***Brevibac* 22.2.4**	***Brevibac* 22.4.13**	***Sacchar* 22.4.25**	***Terribac* 22.2.3**	***Staphylo* 22.3.10**	***Staphylo* 22.3.11**	***Pantoea* 22.4.2**	***Pantoea* 22.3.3**	***Pantoea* 22.4.5**	***Pantoea* 22.4.17**	***Cellulos* 22.3.25**	***Curtob* 22.4.18**
5.55 (775)	Butanediol	1	ND	ND	42.16 (± 42.09)	ND	ND	ND	ND	ND	ND	26.65 (± 11.32)	71.30 (± 25.50)	ND	ND	ND	ND
5.87 (787)	2,3-Butanediol	2	ND	ND	33.32 (± 33.26)	ND	ND	ND	ND	ND	ND	264.24 (± 109.53)	18.22 (± 8.64)	ND	ND	ND	ND
8.72 (898)	Unknown	3	100.85 (± 33.40)	31.26 (± 14.39)	115.57 (± 24.19)	111.69 (± 26.41)	67.07 (± 9.44)	71.18 (± 8.60)	77.94 (± 8.33)	56.69 (± 8.23)	70.79 (± 8.38)	33.45 (± 1082)	25.06 (± 13.15)	99.83 (± 12.12)	64.37 (± 14.12)	72.42 (± 12.60)	39.49 (± 11.98)
10.97 (998)	Octanal^a^	4	15.52 (± 5.40)	13.06 (± 6.23)	13.94 (± 4.95)	14.75 (± 6.42)	16.23 (± 6.68)	18.82 (± 5.52)	16.89 (± 7.02)	16.18 (± 5.44)	23.08 (± 4.95)	12.37 (± 3.99)	22.04 (± 8.66)	41.88 (± 10.42)	19.15 (± 5.40)	30.95 (± 9.03)	23.33 (± 11.27)
11.47 (1024)	1-Hexanol, 2-ethyl	5	1547.00 (± 279.75)	1882.46 (± 355.18)	1417.56 (± 131.82)	1745.95 (± 99.32)	1966.48 (± 189.07)	1774.97 (± 163.54)	1592.28 (± 151.04)	1912.56 (± 263.83)	2122.98 (± 540.49)	697.03 (± 87.74)	653.86 (± 60.82)	613.72 (± 40.57)	413.28 (± 25.32)	1526.52 (± 200.12)	520.70 (± 283.60)
12.79 (1100)	Undecane^a^	6	42.81 (± 8.36)	47.17 (± 2.91)	41.79 (± 4.36)	42.86 (± 5.32)	31.65 (± 4.32)	35.73 (± 3.92)	41.04 (± 4.03)	50.10 (± 6.99)	45.15 (± 7.78)	27.67 (± 1.88)	25.36 (± 3.23)	30.50 (± 2.20)	21.94 (± 1.82)	46.19 (± 5.35)	28.50 (± 4.97)
12.88 (1102)	Nonanal^a^	7	125.02 (± 51.57)	97.84 (± 8.29)	134.56 (± 28.41)	120.36 (± 13.35)	117.51 (± 21.39)	154.57 (± 23.21)	132.41 (± 38.66)	108.67 (± 13.38)	117.05 (± 13.66)	167.52 (± 27.78)	155.22 (± 27.25)	178.97 (± 22.20)	213.19 (± 28.32)	216.45 (± 39.05)	172.05 (± 50.27)
14.03 (1173)	Dimethyl glutarate	8	184.32 (± 168.77)	17.15 (± 7.15)	146.33 (± 36.49)	226.64 (± 38.25)	398.19 (± 189.51)	390.41 (± 74.14)	34.97 (± 22.46)	12.26 (± 12.24)	210.83 (± 22.73)	150.59 (± 75.64)	59.642 (± 38.87)	149.31 (± 17.59)	155.68 (± 25.53)	184.81 (± 51.95)	159.98 (± 17.56)
14.43 (1198)	Dimethyl 2,4-dimethylglutarate	9	283.89 (± 163.12)	134.65 (± 12.18)	278.15 (± 53.26)	367.24 (± 38.86)	566.59 (± 216.13)	529.20 (± 79.45)	160.00 (± 33.56)	136.88 (± 22.89)	324.30 (± 25.99)	236.49 (± 78.58)	125.86 (± 50.68)	239.49 (± 15.57)	246.06 (± 25.30)	320.57 (± 67.42)	257.83 (± 20.21)
14.53 (1204)	Decanal^a^	10	395.49 (± 70.88)	313.90 (± 22.24)	371.17 (± 50.02)	369.84 (± 18.41)	375.68 (± 58.14)	544.48 (± 102.91)	351.02 (± 75.37)	337.21 (± 39.00)	390.471 (± 69.74)	538.96 (± 64.86)	663.32 (± 79.93)	528.99 (± 48.23)	626.63 (± 29.43)	707.07 (± 156.09)	667.60 (± 142.50)
14.74 (1218)	2,5-Dimethylbenzaldehyde	11	263.77 (± 75.83)	110.66 (± 29.59)	8.87 (± 8.86)	54.51 (± 3.89)	82.63 (± 43.12)	684.16 (± 79.00)	194.44 (± 71.37)	78.54 (± 20.84)	385.25 (± 41.04)	46.10 (± 23.03)	11.45 (± 7.18)	36.42 (± 8.36)	22.05 (± 10.62)	391.19 (± 57.95)	26.54 (± 18.68)
15.04 (1238)	2-Methoxy-*p*-cymene	12	102.66 (± 72.51)	6.06 (± 4.82)	48.88 (± 15.53)	90.60 (± 20.59)	201.73 (± 112.75)	189.44 (± 58.11)	12.65 (± 12.63)	2.83 (± 1.73)	82.34 (± 12.35)	68.91 (± 46.60)	25.23 (± 14.56)	53.35 (± 8.04)	56.14 (± 10.47)	87.05 (± 34.50)	79.94 (± 14.05)
15.51 (1270)	Unknown	13	62.43 (± 36.51)	23.91 (± 10.04)	56.80 (± 20.47)	81.65 (± 18.90)	171.02 (± 57.97)	151.21 (± 32.31)	30.91 (± 20.06)	9.84 (± 9.82)	100.61 (± 10.07)	68.21 (± 34.32)	28.44 (± 14.13)	58.79 (± 7.16)	73.63 (± 10.72)	74.90 (± 16.29)	68.53 (± 8.37)
15.61 (1276)	Tridecene	14	18.45 (± 6.91)	16.93 (± 4.42)	15.91 (± 6.59)	39.98 (± 14.22)	22.79 (± 5.83)	50.52 (± 5.00)	48.21 (± 24.82)	20.09 (± 5.76)	29.64 (± 6.61)	20.20 (± 12.43)	15.28 (± 9.56)	67.96 (± 34.71)	32.07 (± 13.18)	20.76 (± 13.26)	27.56 (± 12.12)
15.91 (1300)	Tridecane^a^	15	70.17 (± 13.73)	62.61 (± 20.82)	33.62 (± 20.65)	75.14 (± 5.49)	60.74 (± 15.49)	72.39 (± 7.10)	110.62 (± 24.34)	80.52 (± 5.52)	79.17 (± 12.57)	53.06 (± 14.63)	37.98 (± 16.05)	53.98 (± 9.06)	41.44 (± 11.12)	55.11 (± 14.55)	44.85 (± 12.62)
16.03 (1305)	n-Undecanal	16	69.67 (± 6.92)	49.16 (± 12.42)	60.46 (± 8.91)	70.63 (± 2.62)	67.89 (± 8.49)	98.02 (± 9.11)	105.69 (± 37.85)	57.64 (± 4.12)	68.59 (± 4.80)	71.94 (± 34.88)	105.86 (± 33.98)	85.05 (± 7.89)	124.92 (± 8.22)	124.17 (± 20.91)	76.48 (± 21.90)
16.15 (1314)	Unknown	17	80.84 (± 31.90)	139.54 (± 35.43)	160.65 (± 55.87)	102.51 (± 4.72)	192.91 (± 58.25)	152.08 (± 27.08)	197.44 (± 105.70)	123.34 (± 63.41)	102.02 (± 12.49)	70.64 (± 33.09)	71.52 (± 42.98)	59.11 (± 11.12)	82.00 (± 4.22)	88.34 (± 11.70)	56.01 (± 14.86)
16.61 (1347)	Unknown	18	188.17 (± 157.02)	35.12 (± 16.68)	54.76 (± 24.36)	19.97 (± 8.52)	85.51 (± 27.26)	34.40 (± 5.20)	100.31182 (± 13.71)	24.30 (± 14.09)	39.70 (± 8.20)	24.71 (± 14.61)	16.86 (± 10.79)	29.74 (± 8.59)	21.07 (± 4.95)	29.81 (± 15.78)	11.27 (± 6.22)
16.95 (1372)	3-Hydroxy-2,4,4-trimethylpentyl 2-methylpropanoate	19	21.27 (± 10.01)	18.08 (± 11.75)	12.37 (± 7.94)	41.26 (± 8.51)	65.77 (± 111.65)	16.77 (± 12.56)	146.20 (± 99.72)	45.15 (± 4.64)	40.52 (± 6.99)	14.41 (± 8.96)	9.77 (± 9.76)	23.63 (± 11.44)	16.58 (± 13.22)	18.97 (± 7.91)	7.27 (± 3.99)
17.42 (1406)	Dodecanal	20	102.52 (± 40.24)	68.67 (± 16.86)	90.86 (± 25.07)	86.55 (± 13.51)	79.52 (± 8.05)	151.10 (± 44.93)	118.24 (± 17.58)	91.09 (± 8.77)	118.53 (± 15.61)	140.22 (± 62.25)	131.84 (± 33.89)	80.54 (± 9.98)	165.50 (± 17.86)	149.33 (± 28.86)	110.01 (± 17.85)
17.54 (1316)	Unknown	21	287.83 (± 76.91)	211.86 (± 33.36)	262.79 (± 58.34)	312.61 (± 59.10)	551.82 (± 120.20)	379.74 (± 169.41)	363.02 (± 58.69)	303.53 (± 20.59)	415.05 (± 86.55)	85.86 (± 41.81)	30.74 (± 30.69)	97.00 (± 17.74)	111.92 (± 29.00)	144.98 (± 43.24)	111.96 (± 42.35)
17.91 (1444)	(*E*)-Geranyl acetone	22	311.59 (± 71.00)	152.56 (± 14.29)	195.93 (± 39.99)	321.47 (± 54.46)	521.94 (± 258.91)	441.24 (± 90.07)	1917.28 (± 1728.35)	175.63 (± 57.58)	323.99 (± 49.79)	106.76 (± 38.63)	161.72 (± 23.02)	103.76 (± 40.73)	157.89 (± 18.70)	168.51 (± 50.44)	125.80 (± 44.40)
18.15 (1464)	2,6-di-tert-butyl-*p*-benzoquinone	23	ND	80.60 (± 16.11)	93.25 (± 25.63)	198.97 (± 40.84)	190.73 (± 41.76)	199.50 (± 42.49)	97.24 (± 39.71)	303.80 (± 41.42)	169.44 (± 58.38)	74.27 (± 15.37)	55.59 (± 14.33)	46.74 (± 6.83)	68.21 (± 8.66)	72.23 (± 19.25)	82.79 (± 31.09)
18.23 (1470)	Dodecanol	24	164.67 (± 52.65)	169.76 (± 44.57)	972.13 (± 721.76)	321.31 (± 98.10)	274.79 (± 29.78)	1969.44 (± 771.60)	456.52 (± 76.48)	198.55 (± 18.68)	1334.13 (± 414.80)	1003.34 (± 673.72)	220.96 (± 107.15)	16.71 (± 4.82)	1696.03 (± 555. 9)	461.21 (± 109.00)	331.04 (± 118.37)
18.65 (1500)	Phenol, 2,4-bis(1,1-dimethylethyl)-	25	182.00 (± 42.91)	104.25 (± 42.44)	148.49 (± 43.91)	122.12 (± 32.04)	240.58 (± 83.21)	398.96 (± 103.00)	232.93 (± 71.62)	182.76 (± 35.36)	152.08 (± 11.69)	313.88 (± 164.22)	56.35 (± 17.52)	101.21 (± 14.53)	121.24 (± 13.76)	79.99 (± 12.22)	30.76 (± 17.59)
20.25 (1635)	Benzophenone^a^	26	49.97 (± 8.37)	61.49 (± 12.40)	40.64 (± 7.02)	64.67 (± 7.20)	84.04 (± 3.78)	68.61 (± 1.24)	71.46 (± 19.37)	75.55 (± 6.19)	68.99 (± 8.69)	31.20 (± 4.66)	28.88 (± 3.14)	14.65 (± 4.42)	24.70 (± 2.09)	63.75 (± 8.53)	36.53 (± 12.50)
20.37 (1646)	Methyl dihydrojasmonate	27	72.99 (± 14.80)	74.79 (± 14.63)	69.23 (± 15.14)	27.56 (± 6.40)	58.87 (± 8.45)	47.06 (± 4.92)	42.64 (± 11.95)	98.45 (± 8.67)	91.52 (± 6.77)	9.63 (± 2.13)	1.84 (± 1.84)	3.73 (± 2.29)	4.29 (± 1.17)	49.58 (± 15.50)	27.44 (± 24.19)

^1^Volatile emissions are given in mean peak area divided by 10^4^ with the ± SE reported in brackets. For all treatments five biological replicates were carried out. *ND* compound not detected

^a^Chemical compounds identified using synthetic standards

^b^ID corresponds with the numbers presented in Fig. 2b

## Discussion

In this study, we discovered that bacteria isolated from buckwheat floral nectar affect the foraging behavior of the parasitoid *T*. *basalis* via changes in mVOC composition. To the best of our knowledge, this is the first evidence demonstrating that an insect parasitoid can respond to changes in nectar odors caused by bacteria. From the nectar of buckwheat flowers, we cultured bacteria from three different phyla: Firmicutes (8 isolates), Proteobacteria (4 isolates), and Actinobacteria (2 isolates). The fact that *T*. *basalis* females are attracted to nectar fermented by Firmicutes (*S*. *epidermidis* SAAF 2.3.11 and *T*. *saccharophilus* SAAF 22.2.3), Proteobacteria (*Pantoea* sp. SAAF 22.4.17), and Actinobacteria (*Curtobacterium* sp. SAAF 22.4.18) suggests that parasitoid olfactory responses are not constrained by bacterial phylogeny, although this hypothesis should still be tested in future studies.

Although the number of studies is limited so far, bacterial colonization of nectar has been associated with avoidance of flower visitation by hummingbirds [65], bumblebees [33], and honeybees [27]. Nectar-inhabiting bacteria may be pathogens for pollinators, so avoidance of bacteria-contaminated flowers has been argued to be adaptive [2]. The scenario for insect parasitoids may be different given the fact that parasitoid infection by bacterial pathogens has been rarely recorded, suggesting that parasitoids are not prone to bacterial diseases [17]. This could be the reason why none of the isolates we tested elicited repellence in *T*. *basalis* females. Furthermore, positive effects on parasitoid fitness due to bacteria-mediated effects on floral nectar chemistry have also been documented [40]. For example, the parasitoid *A*. *ervi* increases its longevity when feeding on nectar fermented by *Lactococcus* sp. compared with non-fermented synthetic nectar and this effect has been linked to an increase in the amount of amino acids such as isoleucine, leucine and valine. However, *A*. *ervi* longevity is reduced when feeding on nectar fermented by another bacterial strain, *Asaia* sp. indicating that bacteria-mediated effects on parasitoid fitness are largely dependent on the specific bacterial strain [40]. To clarify whether *T*. *basalis* olfactory responses are adaptive, additional studies are required to investigate if parasitoids are able to gain positive fitness effects when feeding on nectar fermented by the bacterial strains that elicited parasitoid attraction.

From the microbe perspective, it would be interesting to understand if nectar-inhabiting bacteria can obtain benefits from attracting flower-visiting parasitoids. In the case of yeasts, the production of mVOCs that attract insect vectors has been suggested to be advantageous in order to enhance microbial dispersal and thus colonize novel environments [8, 13, 43]. This strategy may be particularly beneficial for yeasts that specialize in floral nectar colonization, such as *Metschnikowia* species, which are believed to be strongly dependent on animal vectors for dispersal [3, 10]. However, whether bacteria may also increase their chances to colonize new environments by attracting insect parasitoids remains to be investigated.

In our study, we found that bacterial fermentation of nectar affects both the qualitative and quantitative composition of nectar odors. Among the qualitative differences, it was found that the mVOCs butanediol and 2,3 butanediol were only detected in the headspace of *Bacillus* sp. SAAF 22.2.27, *P*. *agglomerans* SAAF 22.4.2 and *P. dispersa* SAAF 22.3.3. These microbial volatiles have been shown to have antibacterial properties [16] and thus may give competitive advantages when other microbes colonize the same buckwheat flower. However, the parasitoid *T*. *basalis* seems not to be attracted to those volatiles as the wasps did not show any attraction—at least not in the tested background of synthetic nectar—toward the nectar fermented by the abovementioned isolates. Another mVOC that deserves attention is 2,6-di-tert-butyl-*p*-benzoquinone which was always detected in nectar samples fermented by bacterial isolates, but never in non-fermented control samples. Interestingly, *p*-benzoquinone was detected in the headspace of buckwheat flowers in the study by Foti et al. [22], suggesting that this compound may also be of microbial origin. Antimicrobial activities of *p*-benzoquinone and its derivatives have been reported [47], indicating that production of these compounds, like butanediols, may also provide interspecific competitive advantages to nectar-inhabiting bacteria. However, 2,6-di-tert-butyl-*p*-benzoquinone is not listed among the most relevant compounds in our PLS-DA multivariate statistical analyses, suggesting that it should play a minor role in explaining the olfactory responses displayed by *T*. *basalis*.

In our study, the quantitative effects in nectar odor composition due to bacterial fermentation seem to be important for the olfactory responses of the parasitoid *T*. *basalis*. According to our PLS-DA model, the compounds that have the highest VIP values, and are thus most likely correlated with parasitoid attraction, are 2-methoxy-*p*-cymene, dimethyl glutarate, methyl dihydrojasmonate, and 2,5-dymethylbenzaldehyde. The compounds 2-methoxy-*p*-cymene and dimethyl glutarate have been found in flowers of *Thymus pulegioides* (Lamiales: Lamiaceae) [55] and *Laurus nobilis* (Laurales: Lauraceae) [20], respectively. The compounds dihydrojasmonate and 2,5-dymethylbenzaldehyde have been shown or suggested to play a role in attracting pollinating and parasitoid insects [48, 67]. In particular, 2,5-dimethylbenzaldehyde is present in the flowers of *Rafflesia cantleyi* (Malpighiales: Rafflesiaceae) and possibly involved in the attraction of blowfly pollinators [67]. Methyl dihydrojasmonate is an interesting compound as it has been reported to play a role in parasitoid attraction to plants colonized by the rhizobacterium *Pseudomonas fluorescens* WCS417r when attacked by insect herbivores [48]. In fact, it has been found that *cis*-methyl dihydrojasmonate is one of the main compounds responsible for attracting the parasitoid *Microplitis mediator* (Hymenoptera: Braconidae) toward rhizobacterium-colonized *Arabidopsis thaliana* (Brassicales: Brassicaceae) plants infested with *Mamestra brassicace* (Lepidoptera: Noctuidae) caterpillars [48]. Although it is reasonable to speculate that these four compounds may be involved in *T*. *basalis* attraction toward bacteria-fermented nectar, their synthetic counterparts should be tested in further research to unravel their real contribution to parasitoid olfactory responses [25]. Moreover, it is also possible that parasitoid attraction will depend on blends or specific ratios of these four mVOCs, rather than on a single compound, as has been largely shown for herbivore-induced plant volatiles (HIPVs) and oviposition-induced plant volatiles (OIPVs) [19, 59]. It should also be pointed out that, in nature, VOC emission may be variable and strongly affected by biotic and abiotic factors. For example, a recent study carried out on buckwheat found that drought-stressed plants emit a different composition of flower volatiles due to higher emissions of (*Z*)-3-hexenol, isobutyraldehyde, 2-methylbutanal, and 3-methylbutanal [52].

Our work also contributes to increase the awareness about the need to shift from a bi-partite perspective between flowering plants and parasitoids toward a more comprehensive tripartite plant–insect-microbe perspective [14]. However, research in this field is still at its infancy and several ecological aspects need to be further addressed to understand the impact of nectar-inhabiting microbes for insect parasitoids in more realistic ecological settings. For example, the studies carried out so far, including this work, have tested cell-free nectar fermented by bacteria or yeasts [40, 56], whereas the role played by microorganisms growing naturally on floral nectar has not been considered in the nutritional ecology and foraging behavior of insect parasitoids. Furthermore, investigations have been carried out by inoculating synthetic nectar with only one bacterium at a time, whereas, in nature, nectar bacteria are commonly structured in dynamic microbial consortia [40, 56]. Although we demonstrated that bacteria inhabiting floral nectar can affect the olfactory responses of insect parasitoids, we acknowledge that results may not be easily transferred to field situations.

Finally, our study is also relevant from an applied perspective, particularly for the field of conservation biological control where flowering resources, such as buckwheat, are extensively used to enhance natural enemies’ performances in agro-ecosystems [28, 36]. Since microbes ubiquitously inhabit flowers, where they can modify nectar traits relevant for natural enemies of pests, they should be considered in conservation biological control [14, 41]. So far, plant screening in conservation biological control is mainly based on flowering duration, flower attractiveness, quality, and accessibility of floral nectar [61, 66]. Selecting flowering plants based on their likelihood to host beneficial nectar-inhabiting microbes or carrying out spray applications with bacteria capable of enhancing parasitoid attraction toward flowering resources could be an additional aspect to take into account in conservation biological control programs [41].

## Supplementary Information

Below is the link to the electronic supplementary material.

Supplementary file1 (PDF 233 KB)

## Data Availability

If the manuscript will be accepted, raw data will be archived in the Dryad Digital Repository**.**
