# Supplementary material for: Nectar-Inhabiting Bacteria Affect Olfactory Responses of an Insect Parasitoid by Altering Nectar Odors
Source: Microb Ecol. 2022 Aug 1;86(1):364–76. doi: 10.1007/s00248-022-02078-6 (PMC10293396; doi:10.1007/s00248-022-02078-6)

ELECTRONIC SUPPLEMENTARY MATERIAL

To be submitted to: Microbial Ecology

Nectar-inhabiting bacteria affect olfactory responses of an insect parasitoid by altering nectar odors

Antonino Cusumano[1,2], Patrizia Bella[1,2], Ezio Peri[1,2*], Michael Rostás[3], Salvatore Guarino[4], Bart Lievens[5,6], Stefano Colazza[1,2]

[1]Department of Agricultural, Food and Forest Sciences, University of Palermo Viale delle Scienze, Building 5, 90128 Palermo, Italy. [2]Interuniversity Center for Studies on Bioinspired Agro-Environmental Technology (BATCenter), University of Napoli Federico II, 80055 Portici, Italy. [3]Agricultural Entomology, Department of Crop Sciences, University of Göttingen, Grisebachstr. 6, 37077 Göttingen, Germany. [4]Institute of Biosciences and Bioresources (IBBR), National Research Council of Italy (CNR), Corso Calatafimi 414, Palermo 90129, Italy. [5]Laboratory for Process Microbial Ecology and Bioinspirational Management (PME&BIM), Department of Microbial and Molecular Systems, KU Leuven, Willem De Croylaan 46, 3001 Leuven, Belgium. [6]Leuven Plant Institute (LPI), KU Leuven, 3001 Leuven, Belgium

*correspondance to: Ezio Peri

Supplementary table 1. Bacterial strains recovered from nectar samples of buckwheat flowers, identification based on 16s rRNA gene sequences and GenBank accession numbers

Strain ID	Accession Number[1]	Phylum	Family	Closest match in GenBank [2]	Sequence identity (%)	NCBI Acc. No.
SAAF 22.2.3	ON166782	Firmicutes	Bacillaceae	*Terribacillus saccharophilus*	99.87	NR_041356.1
SAAF 22.2.4	ON166772	Firmicutes	Bacillaceae	*Brevibacterium frigoritolerans*	99.87	MK424281.1
SAAF 22.2.6	ON166770	Firmicutes	Bacillaceae	*Bacillus* sp. (*B. acanthi; B. megaterium; B. aryabhattai; B. flexus; B. zanthoxyli*)	100	MT516450.1; MK508856; MK182804.1; MN326676.1 OL875277.1
SAAF 22.2.27	ON166769	Firmicutes	Bacillaceae	*Bacillus* sp. (*B. subtilis; B. licheniformis; B. paralicheniformis; B. haynesii; B. sonorensis B. glycinifermentas, B. piscis*)	99.87	MK183752.1; MN396732.1; MK559531.1; NR157609.1; MF446619.1, KT005408.1 NR_165685.1
SAAF 22.4.13	ON166769	Firmicutes	Paenibacillaceae	*Brevibacillus* sp. (*B. nitrificans; B. centrosporus*)	100	NR_112926.1; NR_112211.1
SAAF 22.4.25	ON166779	Firmicutes	Paenibacillaceae	*Saccharibacillus endophyticus*	98.61	NR_153708.1
SAAF 22.3.10	ON166780	Firmicutes	Staphylococcaceae	*Staphylococcus hominis*	99.87	MF678883.1
SAAF 22.3.11	ON166781	Firmicutes	Staphylococcaceae	*Staphylococcus epidermidis*	99.87	AP019721.1
SAAF 22.3.3	ON166776	Proteobacteria	Erwiniaceae	*Pantoea dispersa*	99.87	NR_116755.1
SAAF 22.4.2	ON166775	Proteobacteria	Erwiniaceae	*Pantoea agglomerans*	99.75	KY013009.1
SAAF 22.4.5	ON166778	Proteobacteria	Erwiniaceae	*Pantoea* sp. (*P. eucalypti*)	98.74	NR_116112
SAAF 22.4.17	ON166777	Proteobacteria	Erwiniaceae	*Pantoea* sp. (*P. eucalypti*)	98.74	NR_116112
SAAF 22.4.18	ON166774	Actinobacteria	Microbacteriaceae	*Curtobacterium* sp. (*C. pusillum; C. gossypii; C. oceanosedimentum; C. luteum*)	100	LN681569.1; MW466552.1; NR_116064.1; MT781467.1
SAAF 22.3.25	ON166773	Actinobacteria	Promicromonosporaceae	*Cellulosimicrobium* sp. (*C. funkei; C. cellulans*)	99.87	NR_042937.1; NR_115251

[1]GenBank accession numbers of the 16S rRNA gene partial sequences of bacterial stains isolated from buckwheat floral nectar in this study
[2]When BLAST analysis yielded different species with identical scores, all species have been reported in the brackets and the bacterial strain identified at genus level.

Online resource 2. Phylogenetic tree of the bacterial strains isolated from buckwheat nectar samples and a number of reference bacterial strains downloaded from GenBank (Accession numbers provided). The evolutionary history was inferred using the Neighbour-Joining method (Saitou and Nei, 1987). The evolutionary distances were computed using the Jukes-Cantor method. There was a total of about 801 positions in the final dataset. Evolutionary analyses were conducted in MEGA X (Kumar et al., 2018). The percentage of replicate trees in which the associated taxa clustered together in the bootstrap test (1000 replicates) are shown next to the branches. Strains isolated in this study are marked with a triangle.

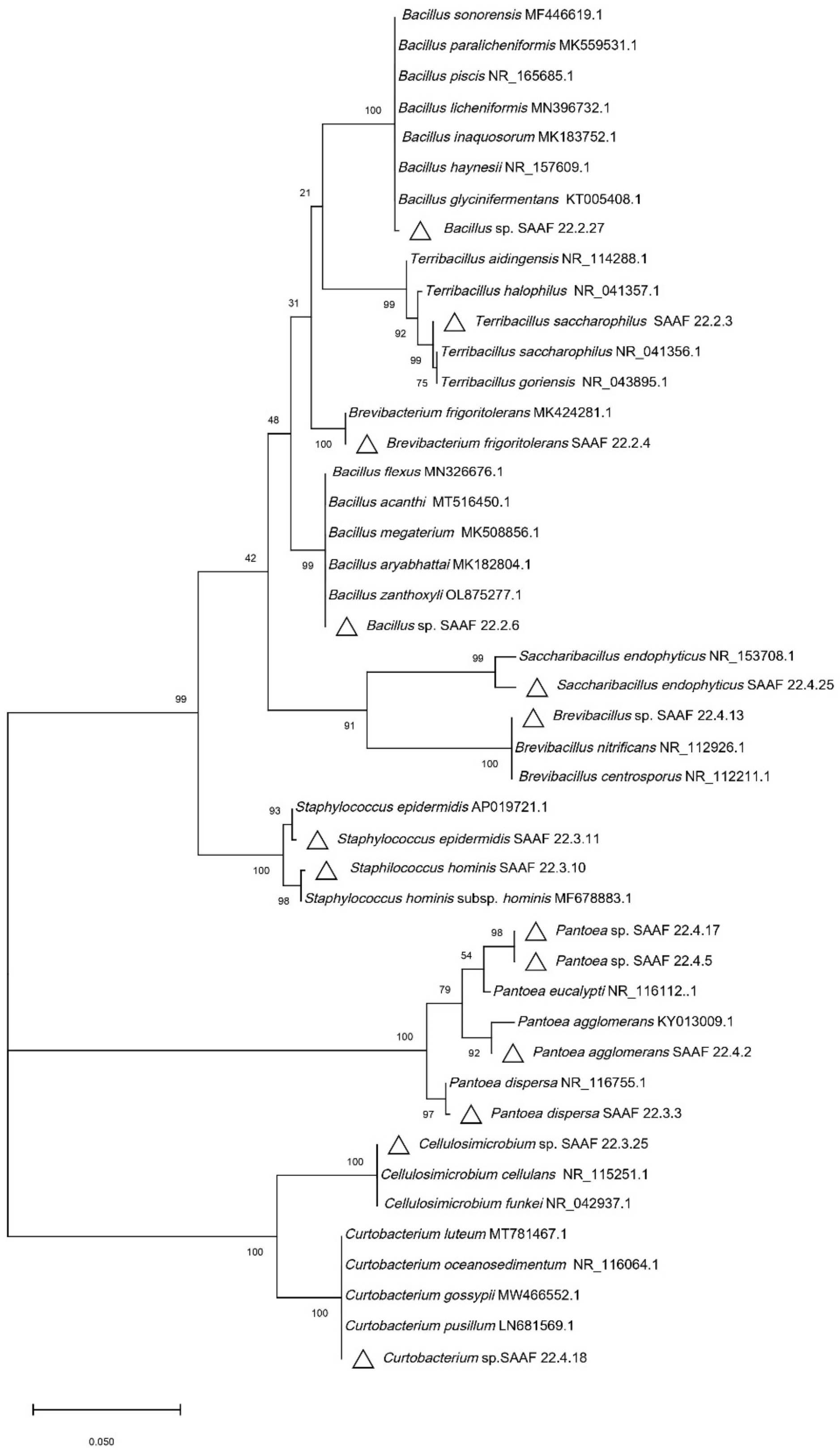

Supplement: Supplementary file 1 — Supplementary file1 (PDF 233 KB) [file 248_2022_2078_MOESM1_ESM.pdf]
